# Supplementary figures and images for: Myeloid cell recruitment versus local proliferation differentiates susceptibility from resistance to filarial infection
Source: eLife. 2018 Jan 4;7:e30947. doi: 10.7554/eLife.30947 (PMC5754202; doi:10.7554/eLife.30947)

**C57BL/6**

**A**

Naïve - Post Birth

Naïve - 18 weeks

Naïve - 23 weeks

Naïve - 25 weeks

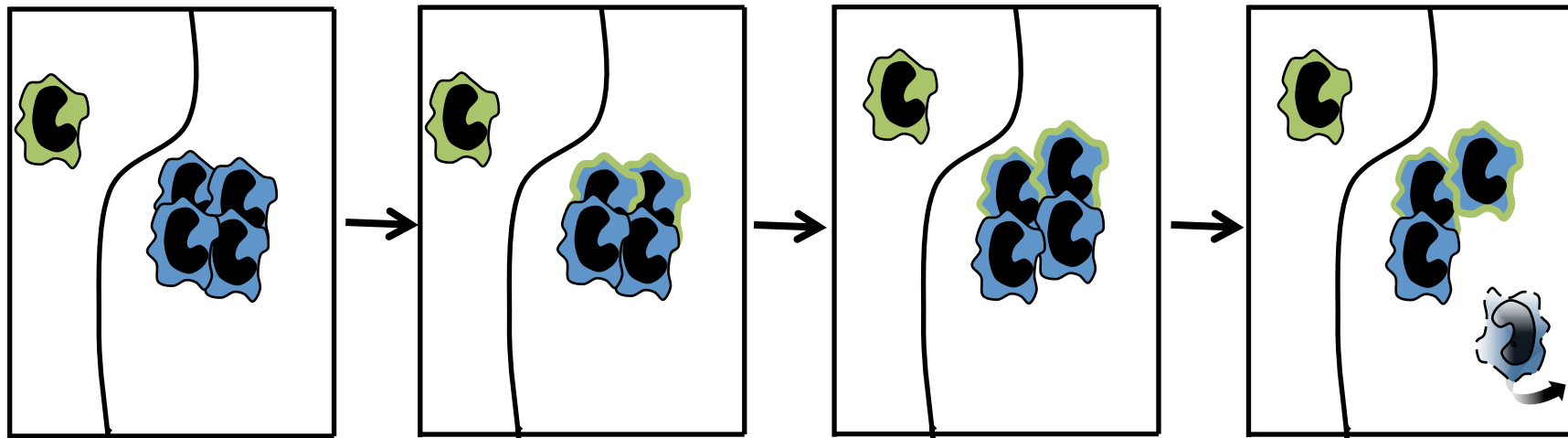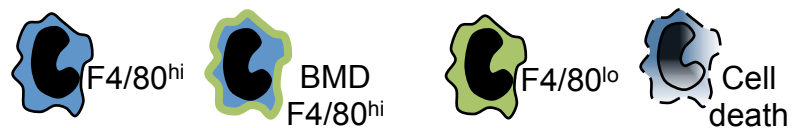

**B**

Infected- Day 35

Infected- Day 50

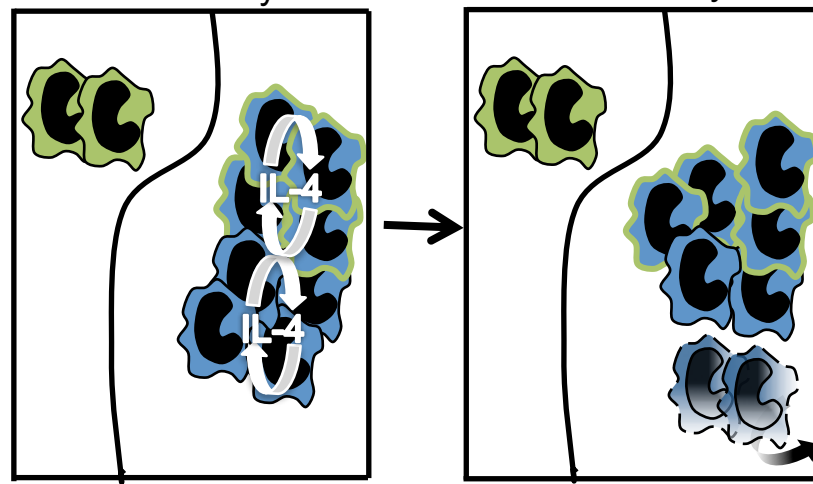

Supplement: Supplementary file 1. — (A) Post birth, prenatally derived F4/80hi resMΦ constitute 80% of the MΦ compartment, by 18–23 weeks of age 50% of the resMΦ compartment has been replenished by bmMΦ which assume residency markers GATA6 and CD102. The degree of bmMΦ contributing to the resMΦ population continues to increase with age, reflective of enhanced proliferative survival of the more recent donor derived resMΦ and eventual death of host derived F4/80hi resMΦ. (B) Upon infection, IL-4 drives proliferation of F4/80hi resMΦ, 50% of which has been derived from bmMΦ by 23 weeks of age, causing expansion of the resMΦ population to 27-fold greater than that of naïve controls. By day 50 pi, the degree of bmMΦ contributing to the resMΦ population has increased further, likely reflecting enhanced proliferative survival of the more recent donor derived resMΦ and eventual death of host derived F4/80hi resMΦ [file elife-30947-supp1.pdf]
